# Regulation of Nitric Oxide Production by δ-Opioid Receptors during Glaucomatous Injury

**DOI:** 10.1371/journal.pone.0110397

**Published:** 2014-10-17

**Authors:** Shahid Husain, Yasir Abdul, Sudha Singh, Anis Ahmad, Mahvash Husain

**Affiliations:** Hewitt Laboratory of the Ola B. Williams Glaucoma Center, Department of Ophthalmology, Storm Eye Institute, Medical University of South Carolina, Charleston, South Carolina, United States of America; Dalhousie University, Canada

## Abstract

To determine the roles of nitric oxide in glaucomatous injury and its regulation by δ-opioid-receptor activation, animals were treated with: 1) a selective inducible nitric oxide synthase (iNOS) inhibitor (aminoguanidine; AG; 25 mg/kg, i.p.); 2) δ-opioid-receptor agonist (SNC-121; 1 mg/kg, i.p.); or 3) with both drugs simultaneously for 7 days, once daily. The loss in retinal ganglion cell (RGC) numbers and their function in glaucomatous eyes were significantly improved in the presence of AG or SNC-121; however, we did not see any significant additive or synergistic effects when animals were treated with both drugs simultaneously. The levels of nitrate-nitrite were significantly increased in the glaucomatous retina when compared with normal retina (normal retina 86±9 vs. glaucomatous retina 174±10 mM/mg protein), which was reduced significantly when animals were treated either with SNC-121 (121±7 mM/mg protein; *P*<0.05) or AG (128±10 mM/mg protein; *P*<0.05). Additionally, SNC-121-mediated reduction in nitrate-nitrite levels was not only blocked by naltrindole (a δ-opioid-receptor antagonist), but naltrindole treatment potentiated the nitrate-nitrite production in glaucomatous retina (235±4 mM/mg protein; *P*<0.001). As expected, naltrindole treatment also fully-blocked SNC-121-mediated retina neuroprotection. The nitrotyrosine level in the glaucomatous retina was also increased, which was significantly reduced in the SNC-121-treated animals. Additionally, the expression level of iNOS was clearly increased over the control levels in the glaucomatous retina and optic nerves, which was also reduced by SNC-121 treatment. In conclusion, our data support the notion that nitric oxide plays a detrimental role during glaucomatous injury and inhibition of nitric oxide production provided RGC neuroprotection. Furthermore, δ-opioid receptor activation regulates the production of nitric oxide via inhibiting the activity of iNOS in the retina and optic nerve.

## Introduction

Glaucoma is a slowly progressing optic neuropathy that causes loss of retinal ganglion cells (RGCs), optic disc changes, and visual-field loss [1], [2]. Currently, intraocular pressure (IOP) lowering strategies are the only effective way to control the disease progression. However, reduction in IOP is not always successful and many patients continue to experience the progressive loss of vision [3], [4]. The mechanisms that lead to the RGC death during disease progression are unclear. However, accumulating literature suggests that multiple mechanisms are involved in RGC death in glaucoma including inflammation [5]–[8], apoptosis [9]–[11], neurotrophic factor deprivation [12], [13], and nitric oxide production [14]–[16]. During the progression of glaucoma, glial cells, mainly astrocytes within the optic nerve head (ONH) [17], also play key roles in the initiation of lesions in glaucomatous optic neuropathy [18]. While astrocytes play a vital role in maintaining a normal physiological state in RGCs, they become activated in response to glaucomatous injury and produce neurotoxic substances like proinflammatory cytokines [7], [8] and nitric oxide which ultimately leads to axonal and RGC loss [14], [15], [19].

Nitric oxide is an important signaling molecule, which regulates various physiological processes in numerous tissues. In the eye it regulates IOP [20], [21], and small quantities could be beneficial to maintain metabolites and blood circulation [22]. However, sustained levels of nitric oxide production may result in direct tissue toxicity and contribute to neuronal degeneration [23]. Nitric oxide is produced by nitric oxide synthase (NOS); three isoforms of NOS have been cloned: constitutive (NOS-1 or nNOS), endothelial (NOS-3 or eNOS), and inducible (iNOS or NOS-2). Inducible NOS has been implicated in several neurodegenerative human diseases including Parkinson and stroke [24]. However, conflicting data have been reported for the role of nitric oxide and nitric oxide synthase in glaucoma-induced retina degeneration and RGC loss [14]–[16], [25], [26].

To clarify the role of nitric oxide in glaucoma pathology we have used a chronic ocular-hypertensive rat glaucoma model [27] and determined if δ-opioid-receptor activation regulates nitric oxide production. Additionally, we determined if δ-opioid-receptor-mediated retina neuroprotection is partly via nitric-oxide-dependent pathways. The data we provide herein support the idea that nitric oxide, mainly produced by iNOS, played a detrimental role during glaucomatous injury, and its inhibition either by its selective inhibitor, aminoguanidine (AG), or by δ-opioid-receptor agonist (SNC-121), provided retina neuroprotection against glaucomatous injury.

## Materials and Methods

### Animals

Adult female Brown Norway rats (3–5 months of age; 150–200 grams; Harlan Laboratories, Inc., Indianapolis, IN, and Charles River laboratory, San Diego, CA) were used in this study. Female rats were chosen because they are calm and easy to handle when measuring IOP in conscious rats. Rats were kept under a cycle of 12-hours light and 12-hours dark for all the studies. Animal handling was performed in accordance with the ARVO Statement for the Use of Animals in Ophthalmic and Vision Research; and the study protocol was approved by the Animal Care and Use Committee at the Medical University of South Carolina.

### Drug Preparation

Delta (δ)-opioid-receptor agonist, SNC-121 (Santa Cruz Biotechnology, Dallas, TX), was dissolved in normal saline (0.9%). SNC-121 (1 mg/kg) was injected intraperitoneally (i.p.) into Brown Norway rats 30 minutes after glaucomatous injury, and treatment was continued for 7 days, once daily. Inducible nitric oxide synthase (iNOS or NOS-2) inhibitor, 25 mg/kg aminoguanidine hydrochloride (AG; Sigma-Aldrich, St. Louis, MO) was also intraperitoneally (i.p.) injected 30 minutes after glaucoma surgery. The AG treatment was continued for 7 days, once daily. In a separate group, animals were treated with both AG and SNC-121 simultaneously for 7 days, once daily. In this group, animals were first treated with AG (25 mg/kg, i.p.) 30 minutes after glaucomatous injury, followed by SNC-121 (1 mg/kg, i.p.) treatment at an interval of 15 minutes. A group of animals was also treated with a selective δ-opioid-receptor antagonist, naltrindole (3 mg/kg; i.p.), 30 minutes after glaucomatous injury, followed by SNC-121 treatment for 7 days, once daily. Drug administration (150–200 µL) was performed daily at the same time between 9 am-11 am. The control group was handled in a similar fashion except that normal saline was injected without SNC-121 or AG.

### Development of Glaucoma Model by Hypertonic Saline Injection

Brown Norway rats (150–200 gm body weight) were housed under a standard 12∶12 (light:dark) cycle. A stable baseline IOP was documented prior to hypertonic saline injection. Rats were anesthetized with ketamine (75 mg/kg) and xylazine (8 mg/kg) and body temperature was maintained at 37°C with a heating pad. Topical proparacaine (0.5%) was applied to the cornea. IOP was elevated using 50 µL of 2 M hypertonic saline into the circumferential limbal vein near the cornea as described earlier [7], [27]. After surgery, an antibacterial ointment (neomycin) was applied at the injection site of each animal to prevent infections. As inclusion criteria following hypertonic saline injection, only animals with an elevated IOP that was at least 25% over baseline were included in the study.

### Pattern Electroretinogram (Pattern-ERG) Recordings

Rats were anesthetized with ketamine (75 mg/kg) and xylazine (8 mg/kg) and body temperature was maintained at 37°C with a heating pad. Pattern-ERG recordings (without dark adaptation) were conducted in both eyes (sequentially) 3 days prior to IOP elevation by hypertonic saline injection, and then weekly post-surgery as described earlier [7], [27]. For the Pattern-ERG amplitudes, measurements were made between a peak and adjacent trough of the waveform as described earlier [27]. Pattern-ERG amplitudes were obtained for each eye by collecting 300 sweeps at an interval of 1 second. The final pattern-ERG amplitude was an average of 300 sweeps for each eye.

### Retinal Ganglion Cell (RGC) Counting

RGCs were visualized and counted by retrograde-labeling using fluorogold as described earlier [7], [27]. Briefly, animals were anesthetized with ketamine (75 mg/kg, i.p.) and xylazine (8 mg/kg, i.p.), and 5% Fluorogold (Fluorochrome, LLC; Denver, CO) was injected into the superior colliculus in animals immobilized in a stereotaxic apparatus. Seven days after injection, animals were euthanized and their eyes were enucleated and fixed in 4% paraformaldehyde for 24 hours at 4°C. After rinsing with PBS, each retina was detached from the eyecup and prepared as a flatmount by mounting with the vitreous side-up. RGCs were counted and averaged per 8 microscopic fields of identical size (150 µm^2^; 20x magnification) per retina by using Image J software (NIH, Bethesda, MD). The automated RGC numbers generated by Image J software were comparable when RGCs were counted manually by two operators in a masked fashion.

### Immunohistochemistry

Optic nerves and eyes of normal and glaucomatous animals were collected at the 7^th^ and 42^nd^ day post glaucomatous injury, as described earlier [7], [27]. Optic nerves and eyes were fixed in paraformaldehyde (4%) overnight at 4°C, then cryoprotected in a 30% sucrose solution overnight at 4°C. Optic nerves and eyes were washed with cold PBS and embedded in OCT compound embedding medium over dry ice. Cryosections of optic nerve and retina were cut in a cryostat at –20°C and tissues were incubated with primary antibody (anti iNOS at 1∶100 dilution; BD BioSciences, San Jose, CA) followed by incubation with FITC-conjugated secondary antibody (1∶400 dilutions; Jackson Immuno Research Laboratories, Inc., West Grove, PA). Negative control slides were incubated with 0.5% BSA instead of primary antibodies. Tissue sections were viewed under a fluorescent microscope and digitized images were captured by a digital camera (Zeiss).

### Western Blotting

An equivalent amount of retina lysate (15 µg/lane) was loaded onto 10% SDS-PAGE. Proteins were separated and transferred to nitrocellulose membrane. The membranes were blocked with 5% nonfat dry milk, and then incubated with appropriate primary antibody (anti-Nitrotyrosine at a 1∶1000 dilution; anti-iNOS at a 1∶1000 dilution; or anti-β-actin at a 1∶3000 dilution) for 12 hours at 4°C. After washing, membranes were incubated with appropriate secondary antibodies (HRP-conjugated at 1∶3000 dilution). The membranes were treated with enhanced chemiluminescent reagent to detect the signals using a Biorad Versadoc imaging system (Biorad, Hercules, CA).

### Measurements of Nitrate-Nitrite in Retina Extracts

Nitrate-nitrite was estimated by using “Cayman’s Assay Kit” (Cayman, Item number-780001, Ann Arbor, MI). This method involves a two-step process. Briefly, the retinas were homogenized and centrifuged at 10,000 *g* for 10 minutes. The supernatant was collected to measure nitrates-nitrites. The reaction was started by adding 80 µL of retina samples, 10 µL of enzyme cofactor, and 10 µL nitrate reductase in a 96-well plate. After 1 hour, 50 µL Greiss reagents were added and allowed to incubate for 30 minutes. The changes in absorbance were measured at 550 nm. The amount of nitrate-nitrite was expressed as mM of nitrate-nitrite per milligram protein.

### Statistical Analysis

The data are expressed as mean ± SEM. The comparisons were made using Student *t* test for paired samples or ANOVA with Bonferroni post test for multiple comparisons (Graph Pad Software, Inc., San Diego, CA). A *P* value of <0.05 is considered significant.

## Results

**Figure 1:**
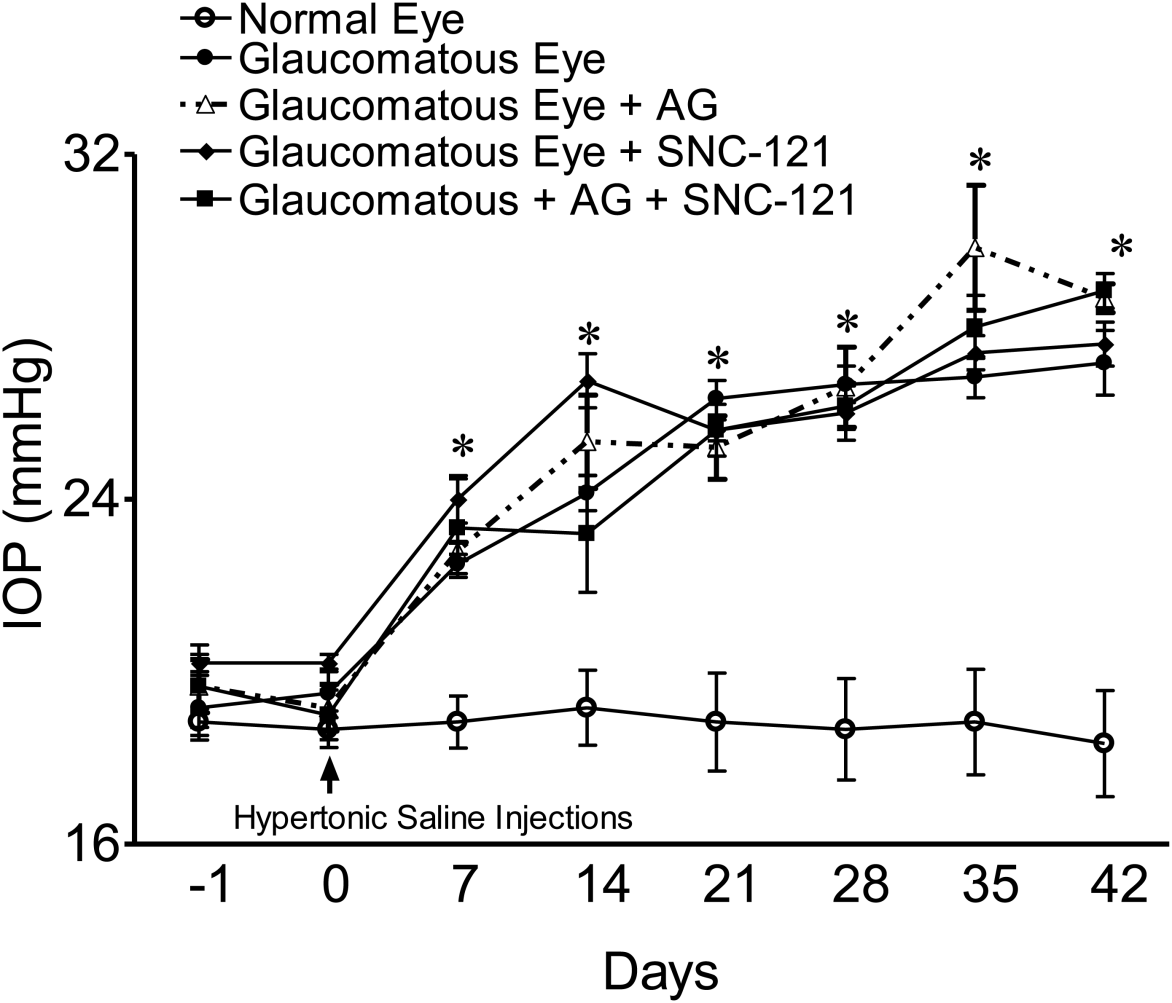
Intraocular pressure (IOP) measurements in normal and glaucomatous eyes. IOP of normal eyes and eyes from a chronic glaucoma model with and without AG (25 mg/kg; i.p.) and SNC-121 (1 mg/kg) treatment for 7 days (once daily) was measured as described in the Methods. IOP was elevated in one eye of Brown Norway rats by injecting approximately 50 µL of 2.0 M hypertonic saline, while the contralateral eye served as the control. Rats were maintained for up to 6 weeks post-surgery. Data are mean ± SE. *P<0.05; n = 6–8 for each group.

**Figure 2:**
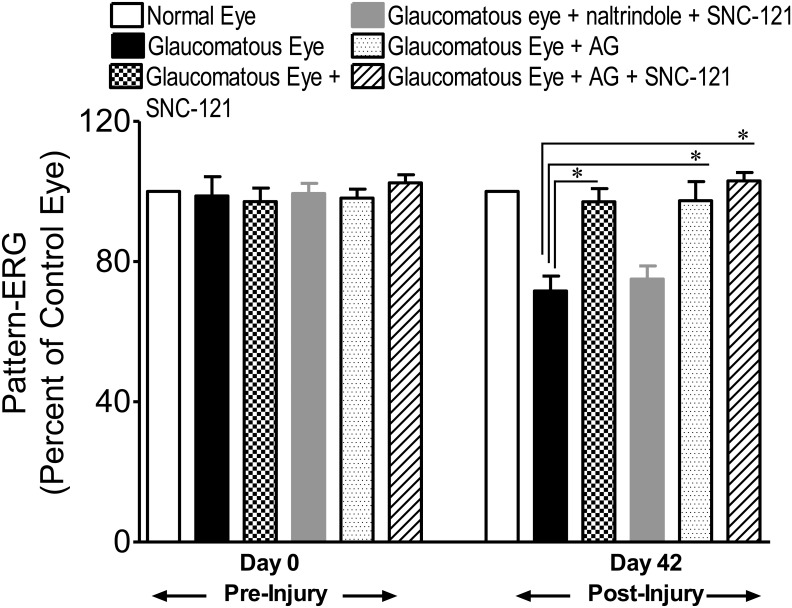
Pattern ERG recordings in normal and glaucomatous eyes. Changes observed in Pattern ERGs of untreated, AG or SNC-121-treated glaucomatous rat eyes are shown as a percentage of the contralateral control eye. In these experiments, Brown Norway rats were treated with AG (25 mg/kg, i.p.) or SNC-121 (1 mg/kg, i.p.), individually or together simultaneously, 30 minutes after hypertonic saline injections. Animals were treated with naltrindole 30 minutes after glaucomatous injury followed by SNC-121 treatment. The drug treatment was continued once daily for 7 days in all groups. Data are mean ± SE; *P<0.05; n = 7–8.

Intraocular pressure (IOP) was raised by injecting 2 M hypertonic saline into limbal veins and IOP was measured up to 42 days at an interval of 7 days, as described earlier [7]. A significant elevation in IOP was seen as early as 7 days post surgery and was maintained up to 42 days following IOP elevation. Similar to our previous studies, we did not see any significant changes in IOP of normal and glaucomatous eyes when animals were treated with a selective δ-opioid-receptor agonist, SNC-121 (1 mg/Kg, i.p.), for 7 days [7] (**Figure 1**). To determine the effects of aminoguanidine (AG) alone or in combination with SNC-121 on IOP, animals were either treated with AG (25 mg/kg, i.p.) alone for 7 days, once daily, or with AG (25 mg/kg, i.p.) and SNC-121 (1 mg/Kg, i.p.) simultaneously for 7 days, once daily. As shown in **Figure 1**, elevated IOP in glaucomatous animals was not changed significantly when animals were treated either with AG or AG plus SNC-121 simultaneously. The functional response of retinal ganglion cells (RGCs) in normal and glaucomatous eyes was measured by pattern-electroretinogram (Pattern-ERG) at 42^nd^ day, post glaucomatous injury. To determine if δ-opioid-receptor-mediated retina neuroprotection was partly mediated via nitric oxide-dependent pathways, animals were treated with a selective iNOS inhibitor (AG; 25 mg/kg, i.p.), a selective δ-opioid-receptor agonist (SNC-121; 1 mg/kg, i.p.) individually or with both drugs simultaneously for 7 days, once daily. As shown in **Figure 2**, the Pattern-ERG amplitudes were reduced by 35% in glaucomatous animals (control eyes 100±00% vs. glaucomatous eyes 65±6%; *P*<0.05). Treatment of animals with AG for 7 days provided retina neuroprotection against glaucomatous injury (glaucomatous eyes, 65±6% vs. glaucomatous eyes + AG, 93±6%; *P*<0.05). Similar to our previous findings [7], SNC-121 treatment also provided a significant amount of retina neuroprotection; however, we did not see any significant additive or synergistic effects when animals were treated with both drugs (e.g., AG and SNC-121) simultaneously (glaucomatous eyes, 65±6% vs. glaucomatous eyes + AG + SNC-121, 110±8%; *P*<0.05). As expected, SNC-121-mediated retina neuroprotection was fully-blocked by pretreatment with δ-opioid-receptor antagonist, naltrindole (**Fig. 2**).

**Figure 3:**
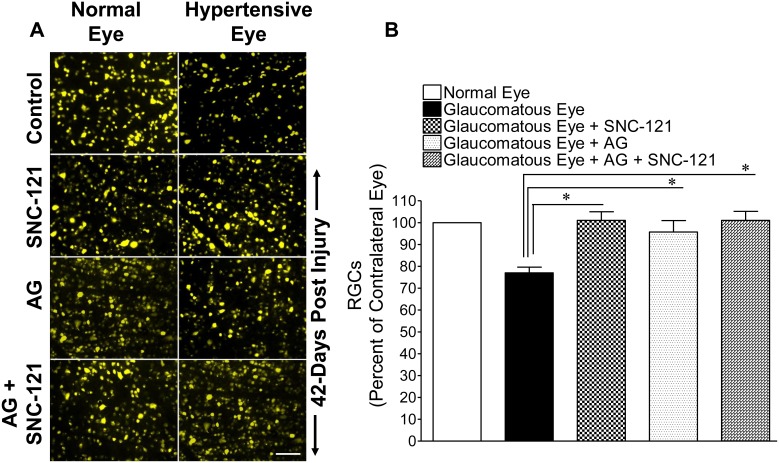
Retina flat mounts of normal and glaucomatous eyes. Fluorescence micrographs of flatmounted retinas depicting Fluorogold-labeled retina ganglion cells (RGCs) in normal and glaucomatous eyes in the absence or presence of drugs (**A**). Briefly, 3 µL of a 5% solution of Fluorogold was injected into the superior colliculus of anesthetized animals. Seven days post injection, animals were euthanized and retinas were prepared as flatmounts, vitreous-side facing up. Fluorescent RGCs were visualized under Zeiss microscopy. Bar = 20 µm. Total RGC tallies in glaucomatous eyes in the absence or presence of drugs. RGCs were counted in 8 microscopic fields of identical size (150 µm^2^ area) for each retina, using Image J software (**B**). *P<0.05; n = 6 for each group.

To confirm that declines in Pattern-ERG amplitudes were due to the loss of RGCs, they were visualized by retrograde-labeling with bilateral injections of fluorogold into the superior colliculus (**Fig. 3A**). The loss of RGCs in the glaucomatous eyes was reduced when animals were treated either with AG (25 mg/kg; i.p.) or SNC-121 (1 mg/kg; i.p.) for 7 days; however, we did not see any further decline in the rate of RGC loss when animals were treated with both drugs simultaneously. Quantification of RGCs in normal eyes, glaucomatous eyes, AG-treated glaucomatous eyes, SNC-121-treated glaucomatous eyes, or both drug-treated glaucomatous eyes are shown in **Figure 3B**. The mean number (± SE) of fluorogold-positive RGCs were: normal eyes, 2150±99 RGCs/mm^2^; glaucomatous eyes, 1666±105 RGCs/mm^2^ (23% less than normal eyes; *P*<0.05); AG-treated glaucomatous eyes, 2012±108.1 RGCs/mm^2^ (21% greater than the glaucomatous eyes; *P*<0.05), SNC-121-treated glaucomatous eyes, 2223±75 RGCs/mm^2^ (33% greater than the glaucomatous eyes; *P*<0.05), and AG + SNC-121-treated glaucomatous eyes, 2181±94.59 RGCs/mm^2^ (31% greater than the glaucomatous eyes; *P*<0.05).

**Figure 4:**
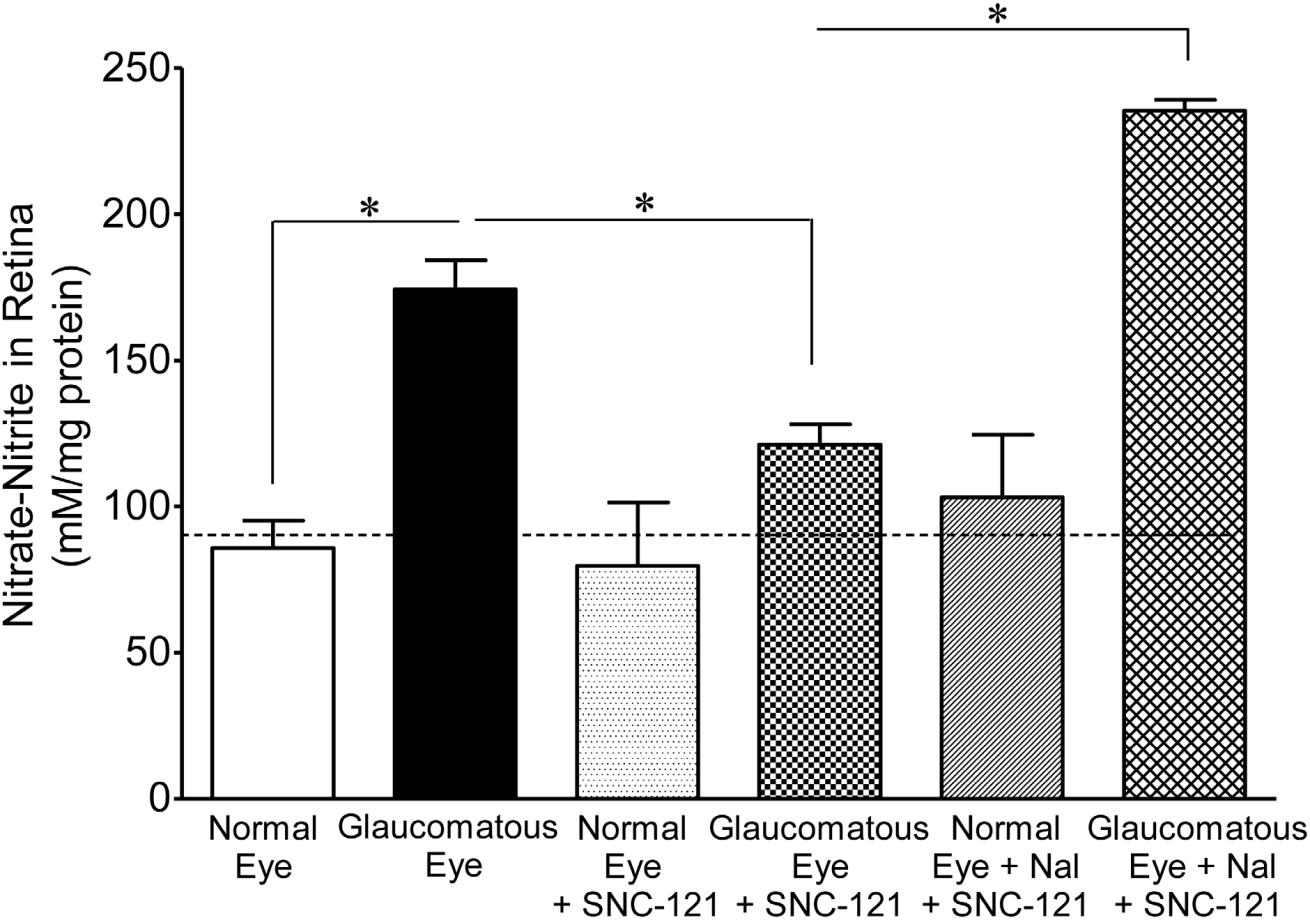
Measurements of nitrate-nitrite in normal and glaucomatous eyes in the presence of SNC-121. Animals were treated with SNC-121 (1 mg/kg, i.p.) 30 minutes after glaucomatous injury, and continued once daily for 7 days. A separate group of animals was pretreated with naltrindole (Nal; 3 mg/kg, i.p.) followed by SNC-121 (1 mg/kg, i.p.) treatment 30 minutes after glaucomatous injury, and continued for 7 days, once daily. The absorbance was read at 550 nm and absorbance was plotted against the nitrate standards. The amount of nitrate-nitrite is expressed as millimolar nitrates-nitrites per milligram protein. Data are expressed as mean ± SEM, (*P<0.05; n  = 4–7).

**Figure 5:**
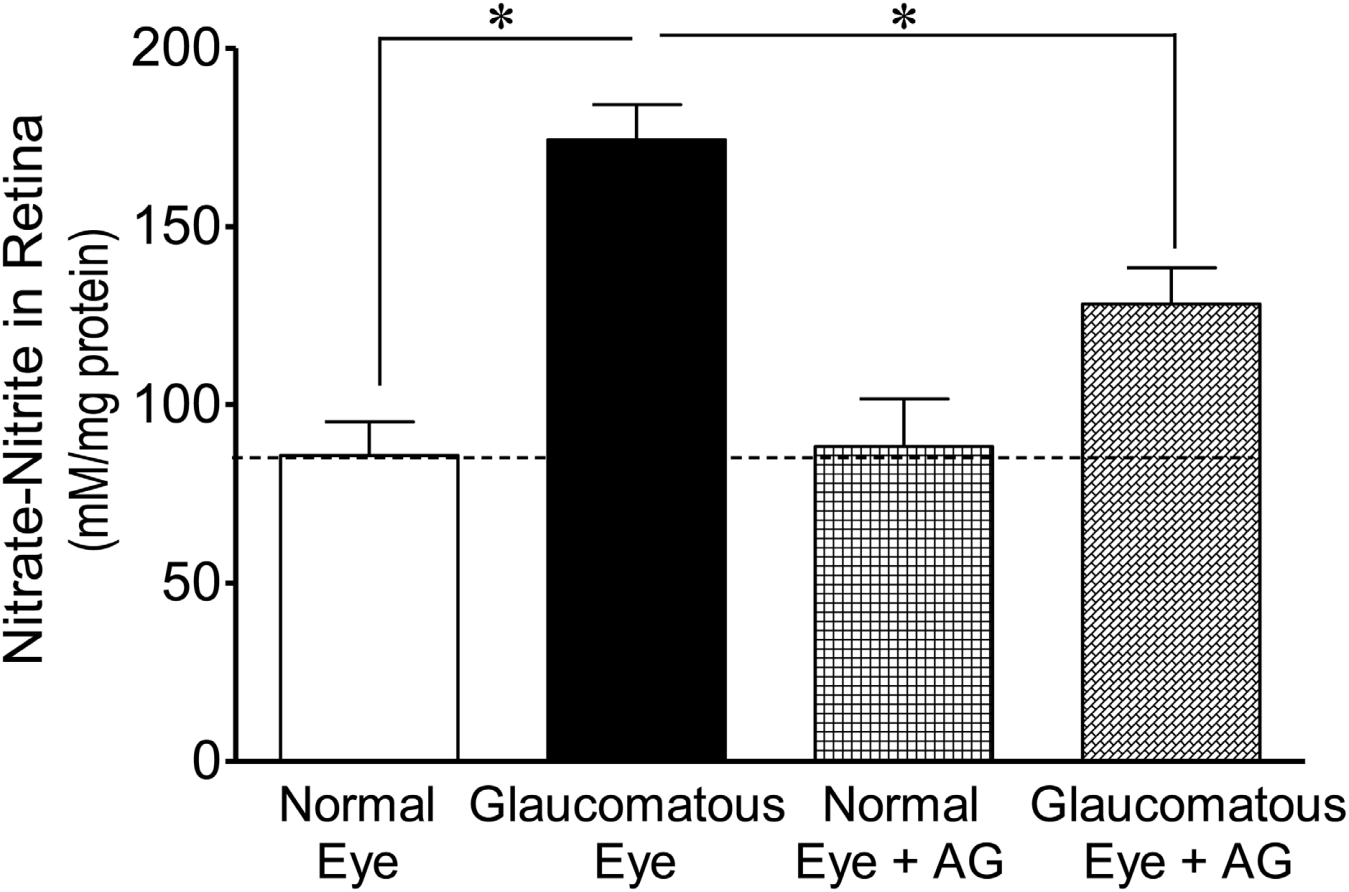
Measurements of nitrate-nitrite in normal and glaucomatous eyes in the presence of AG. Animals were treated with AG (25 mg/kg, i.p.) 30 minutes after glaucomatous injury and continued for 7 days, once daily. The absorbance was measured at 550 nm and absorbance was plotted against the nitrate standards. The amount of nitrate-nitrite is expressed as millimolar nitrates-nitrites per milligram protein. Data are expressed as mean ± SEM, (*P<0.05; n = 4–6).

**Figure 6:**
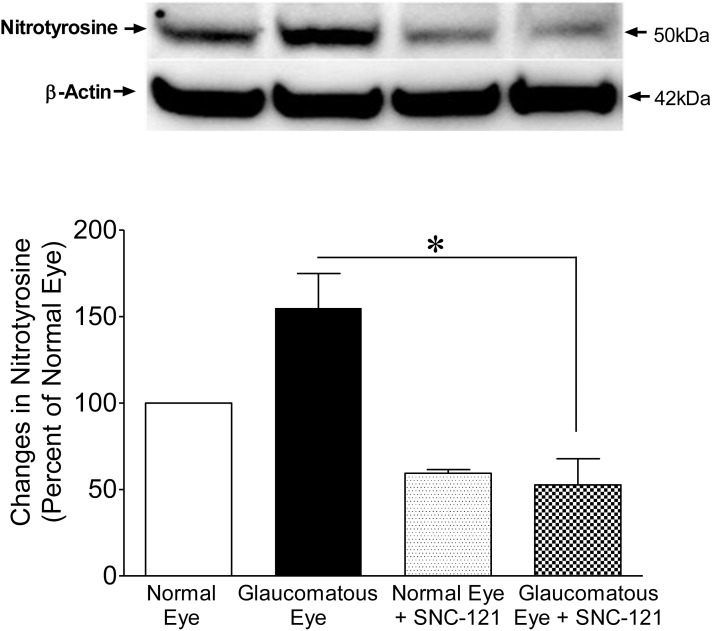
Measurement of protein nitrosylation in retina by Western blotting using nitrotyrosine antibodies in response to glaucomatous injury. Animals were treated with SNC-121 (1 mg/kg, i.p.) 30 minutes after glaucomatous injury and continued for 7 days, once daily. Retina extracts were collected at the 7^th^ day, post injury, and analyzed using anti-nitrotyrosine antibodies and appropriate secondary antibodies (HRP-conjugated; dilution 1∶3000). The signal was captured using enhanced chemiluminescent reagent and the Biorad Versadoc imaging system. Data shown are representative of four independent experiments. Data are expressed as mean ± SEM. *P<0.05; n = 4.

To dissect out the molecular mechanisms, we measured the nitrate and nitrite levels, which are the end products of nitric oxide metabolism. We believe that nitric oxide is produced in an early stage of glaucoma; so we collected whole retina extracts to measure nitrates and nitrites at the 7^th^ day, post glaucomatous injury. The levels of nitrate-nitrite were significantly increased in the retina sample of glaucomatous eyes when compared with normal eyes (normal eyes, 86±9 vs. glaucomatous eyes, 174±10 mM/mg protein; *P*<0.001; **Figs. 4** **–** **5**). The levels of nitrate-nitrite were significantly reduced in glaucomatous eyes when animals were treated either with SNC-121 (121±7 mM/mg protein; *P*<0.05; **Fig. 4**) or AG (128±10 mM/mg protein; *P*<0.05; **Fig. 5**) for 7 days, once a day. To confirm that SNC-121-mediated reduction in nitrate-nitrite levels was via activation of δ-opioid receptors, animals were treated with a selective δ-opioid-receptor antagonist, naltrindole. As shown in **Figure 4**, SNC-121-mediated reduction in nitrate-nitrite levels was not only blocked, but naltrindole treatment potentiated the nitrate-nitrite production in glaucomatous eyes (235±4 mM/mg protein; *P*<0.001; **Fig. 4**). Surprisingly, naltrindole increased the basal levels of nitrate-nitrite in normal eyes, although this change was not statistically significant. Nitric oxide is a versatile free-radical that mediates numerous biological functions. Although nitric oxide can modify numerous signaling pathways, the reaction of nitric oxide with cysteine residues in proteins, a process known as nitrosylation, is emerging as one of the most important mechanisms. This post-translational modification, *S*-nitrosylation, is a physiologically-important event that affects a wide variety of proteins involved in a number of cellular processes. As shown in **Figure 6**, an increase in the nitrotyrosine level in the retina sample of glaucomatous eyes was seen when compared with the normal eye on the 7^th^ day, post injury. Interestingly, the nitrotyrosine levels were reduced in SNC-121-treated animals (**Fig. 6**).

**Figure 7:**
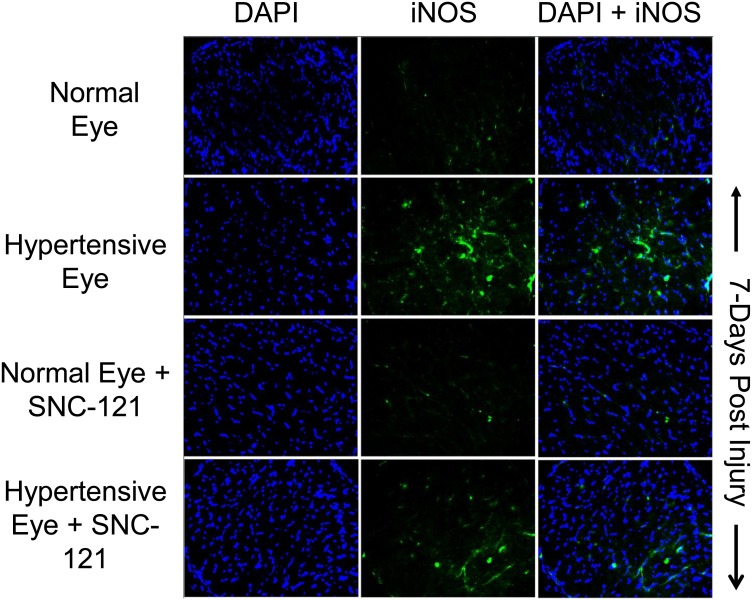
Changes in iNOS expression in glaucomatous optic nerve at 7 days, post injury. The optic nerve (non-myelinated; 2 mm post globe) of Brown Norway rats was removed 7-days post glaucomatous injury. Contralateral optic nerve was used as the normal control. Cryosections were immunostained by anti-iNOS antibodies as indicated horizontally. Ocular treatments are indicated vertically. Green color indicates staining for iNOS and blue nuclei for DAPI. There was no positive staining when primary antibodies were omitted (data not shown). Data shown here are representations of at least four independent experiments. A total of 10 animals were used in this experiment. Comparable staining for iNOS was seen in at least 4 animals in each treatment group.

To further confirm that elevation in nitric oxide production during glaucomatous injury is due to iNOS up-regulation and/or activation**,** we measured the iNOS levels in optic nerve at 7 and 42 days, post injury. As shown in **Figures 7** **–** **8**, the expression levels of iNOS were clearly increased over control levels in glaucomatous optic nerves, which was fully-attenuated in SNC-121-treated glaucomatous optic nerve. However, the iNOS expression pattern was not changed by SNC-121 treatment in normal optic nerves. Additionally, expression of iNOS was increased in the retina on day 7^th^ and 42^nd^, post glaucomatous injury; which was significantly reduced by SNC-121 treatment (**Fig. 9**).

**Figure 8:**
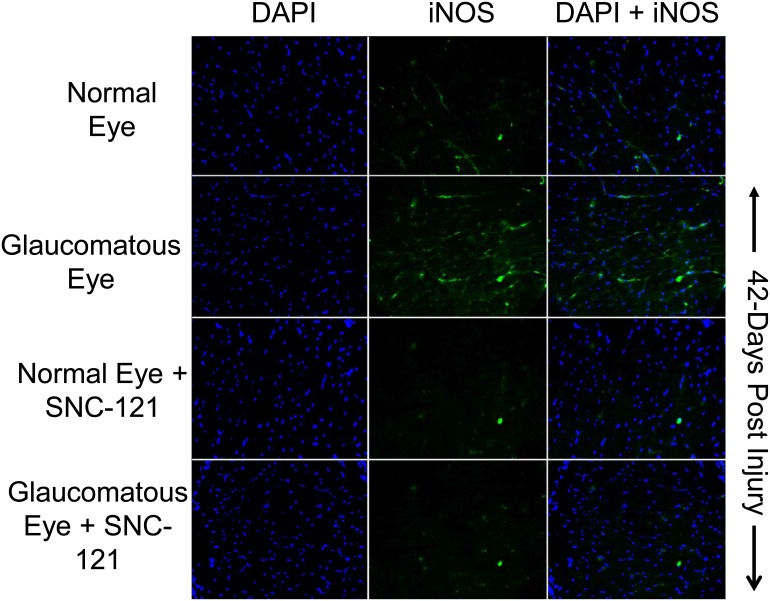
Changes in iNOS expression in glaucomatous optic nerve at 42 days, post injury. The optic nerve (non-myelinated; 2 mm post globe) of Brown Norway rats was removed 42-days post glaucomatous injury. Contralateral optic nerve was used as the normal control. Cryosections were immunostained by anti-iNOS antibodies as indicated horizontally. Ocular treatments are indicated vertically. Data shown in this Figure are a representation of at least four independent experiments. A total of 10 animals were used in this experiment. Comparable staining for iNOS was seen in at least 4 animals in each treatment group.

**Figure 9:**
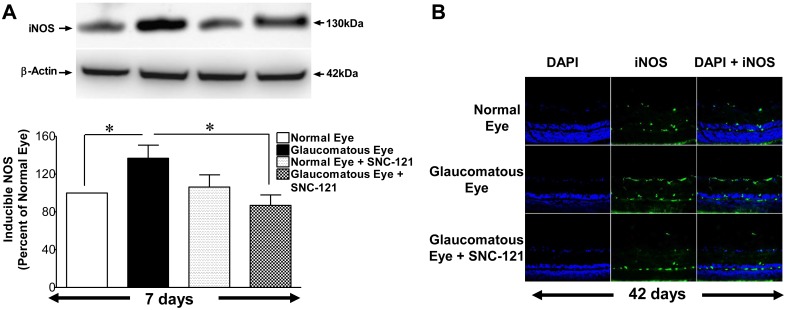
Changes in iNOS expression in glaucomatous retina at 7 and 42 days, post injury. (A) Brown Norway rats were treated with SNC-121 (1 mg/kg, i.p.) 30 minutes after glaucomatous injury and continued for 7 days, once daily. Retina extracts were collected at the 7^th^ day, post injury, and analyzed using anti-iNOS antibodies and appropriate secondary antibodies (HRP-conjugated; dilution 1∶3000). The signal was captured using enhanced chemiluminescent reagent and the Biorad Versadoc imaging system. Data are expressed as mean ± SEM. *P<0.05; n = 4. (B) The eyes of Brown Norway rats were removed 42-days post glaucomatous injury. Cryosections of retina were immunostained by anti-iNOS antibodies as indicated horizontally. Ocular treatments are indicated vertically. Data shown in this Figure are a representation of at least four independent experiments.

## Discussion

We determined that activation of δ-opioid receptors by exogenous ligand (SNC-121) blocked nitric oxide production and subsequently rescued retinal ganglion cells (RGCs) from glaucomatous injury. Nitric oxide at physiological concentrations controls vascular inflammation and vascular injury by inhibiting the proinflammatory gene expression, but excessive production of nitric oxide can cause tissue toxicity and cell death [24]. Although the pathological role of nitric oxide in several neurodegenerative human diseases is somewhat clear, the pathological role of nitric oxide in glaucoma remains in question. For example: an increase in the inducible nitric oxide synthase (iNOS) has been reported in astrocytes and the optic nerve head of primary open-angle glaucoma (POAG) patients [14], [16], [28]. Furthermore, aminoguanidine (AG), a selective iNOS inhibitor, treatment provides RGC neuroprotection in an extraocular vein cauterization glaucoma model [15]. There was a 36% loss in RGCs in this cautery glaucoma model which was reduced to 9.6% by 60 mg/kg AG treatment. In non-glaucoma models, nitric oxide is produced mainly by iNOS that has been implicated in ischemia-induced retinal degeneration [29], [30]. The iNOS inhibition resulted in the RGC neuroprotection against ischemia [31] and axotomy [32]. In contrast, studies also have shown that iNOS levels are not changed in POAG patients [26] and iNOS does not play a detrimental role in glaucomatous injury in chronically-elevated IOP glaucoma model (i.e., a model of aqueous humor outflow obstruction). Moreover, iNOS expression was not correlated with the severity of optic nerve damage [26]. Additional studies have shown that iNOS does not play a detrimental role in optic neuropathy in the DBA/2J glaucoma model [25]. In view of these conflicting results obtained from different laboratories using different experimental glaucoma models, we decided to further test the potential involvement of nitric oxide and inducible NOS (iNOS) in glaucomatous injury. Additionally, we determined if δ-opioid-receptor activation regulates the nitric oxide production.

Although, contradictory reports have been presented in the literature for RGC and optic nerve neuroprotection by iNOS inhibitor (e.g., AG), but all the studies were agreed that AG treatment does not change IOP in normal and glaucomatous animals [15], [26]. Our data is in agreement with previous reports showing that AG treatment had no significant effect on IOP (**Fig. 1**). Additionally, our data presented herein clearly demonstrated that blocking nitric oxide either directly by AG or indirectly via activating δ-opioid-receptors by SNC-121, provided RGC neuroprotection. The functional outcome (Pattern-ERG) of the retina is also changed in the δ-opioid-receptor agonist-treated animals, suggesting that SNC-121 possibly crosses the blood retinal barrier. The most striking findings were that iNOS production was not only elevated in the retina, but also increased in the optic nerve in response to glaucomatous injury, which was attenuated in the presence of SNC-121. Additionally, we have shown that changes in iNOS expression are occurring at an early stage of glaucoma progression (day 7), while other studies [15], [26] have measured such changes in iNOS expression at 6–9 months, post glaucomatous injury. Our results confirmed that the enhanced levels of nitric oxide seen in glaucomatous eyes were due to up-regulation of iNOS not only in the retina, but also in the optic nerve, which may be playing a crucial role in orchestrating RGC death in a paracrine fashion during the pathogenesis of glaucoma.

Our findings are consistent with Shareef and Neufeld [15], [16] who reported that treatment of glaucomatous rats with AG provide retina neuroprotection. However, our results do not agree with Pang and colleagues [26] while both studies have used a similar glaucoma rat model. However, there were numerous procedural differences in the two studies (e.g., Pang [26] and current study) and the discrepancies seen in these studies could be related to such variation in the protocols. For example: 1) normal and glaucomatous animals were kept under constant dim light that resulted in a higher baseline IOP (28 mmHg) versus dark-light cycle with baseline IOP (18–21 mmHg) in our studies; 2) pretreatment of animals with AG for 7 days prior to glaucomatous injury versus treatment of animals 30 minutes post glaucomatous injury in our studies; 3) duration of AG treatment in drinking water for 42 days versus AG treatment (intraperitoneally) for 7 days in our studies, 4) dosage differences of AG; and 5) the lack of RGC function measurement versus Pattern-ERG measurements in our studies. At this time, we cannot explain why genetic ablation of iNOS is not neuroprotective in the DBA/2J model. We believe, this area of research is still not fully-developed and more work is needed to understand a broader role of nitric oxide in general, and iNOS in particular for glaucoma pathology. Overall, our IHC data in retina and optic nerve, Western blotting data, and functional data (Pattern-ERG) support a neurodegenerative role of iNOS during glaucomatous injury.

Recently, we have shown that activation of δ-opioid receptors by a selective agonist (SNC-121) provides retina neuroprotection against glaucomatous injury via attenuation of p38 MAP kinase activity and TNF-α production [7]. Studies have shown that δ- and κ-opioid-receptor-activation provides brain neuroprotection via nitric oxide regulation [33], [34]. The role of nitric oxide in δ-opioid-mediated retinal neuroprotection against glaucomatous injury has not been defined. To determine the roles of iNOS in δ-opioid-receptor-mediated retina neuroprotection, we have treated animals with AG and SNC-121 after glaucomatous injury. We did not see any additional beneficial effects on RGC neuroprotection when animals were simultaneously-treated with AG and SNC-121, suggesting that SNC-121 treatment fully blocked the activity of iNOS. To further confirm the roles of δ-opioid-receptor-activation in iNOS regulation during glaucomatous injury, we measured the production of nitrates and nitrites in the presence or absence of SNC-121. It is very difficult to measure appreciable levels of nitric oxide due to its short life; however nitrites are the stable reservoir that can reduced to bioactive nitric oxide and other nitrogen reactive species during hypoxic conditions [35]. Both AG and SNC-121 treatment reduced the amount of nitrates-nitrites significantly in the glaucomatous eyes. As expected, SNC-121-mediated reduction in nitrates-nitrites was reversed by the δ-opioid-receptor antagonist, naltrindole. These data suggested that SNC-121-mediated reduction in nitrates-nitrites was due to δ-opioid-receptor-activation. It is important to emphasize that naltrindole treatment potentiated the levels of nitrate-nitrite production in both normal and glaucomatous retinas, suggesting that an endogenous tone of δ-opioid-receptor-activation is required to control endogenous levels of nitric oxide.

Nitrotyrosine is identified as an indicator or marker of cell damage, inflammation, and nitric oxide production. In general, oxidative stress increases the production of superoxide (O_2_^−^) and nitric oxide forming ONOO^−^ (peroxynitrite). Under pathophysiological conditions, iNOS synthesizes excessive quantities of nitric oxide, which combines chemically with superoxide to form the highly reactive and destructive peroxynitrite [36]. Peroxynitrite causes the nitrosylation of cellular proteins, lipids, and DNA, which triggers apoptosis and ultimately cell death [37]. It is difficult to determine the production of peroxynitrite, usually nitrotyrosination of proteins is a detectable marker for peroxynitrite^−^. Our data demonstrate that nitrotyrosination was increased in response to glaucomatous injury and δ-opioid-receptor-activation significantly reduced the nitrotyrosination in glaucomatous eyes.

In summary, the findings of the present study support the notion that nitric oxide plays a detrimental role during glaucomatous injury and inhibition of nitric oxide provides RGC neuroprotection. Furthermore, the activation of δ-opioid receptors regulates the iNOS activity/expression that will lead to the attenuation of nitric oxide production and rescue RGCs from glaucomatous injury.
